# Proceedings of the Buffalo Medical and Surgical Association

**Published:** 1888-09

**Authors:** W. H. Bergtold

**Affiliations:** Secretary


### *PROCEEDINGS OF THE BUFFALO MEDICAL AND SURGICAL ASSOCIATION.*

Reported by W. H. BERGTOLD, M. D., Secretary.

Regular monthly meeting, held August 7th, in the Buffalo Library Building; President VAN PEYMA in the chair.

After the reading and approval of the minutes, Dr. W. H. HEATH exhibited an interesting monstrosity. The mother presented no unusual features or history during her confinement, save her inordinate girth. There were present, to account for this abnormality, two factors, viz., an exceedingly large placenta and a great accumulation of anniotic fluid. By casual examination, the monster appeared to be anencephalous, while closer scrutiny proved that it was really a case of *spina bifida*, the laminar portions of the cervical vertebræ being deficient. A well-developed cranium and face existed, with, however, a peculiar implantation of the head on the trunk, causing the child to appear anencephalous.

Discussion:

DR. MYNTER was reminded by the case that he had never met with an example of spina bifida until the previous week.

DR. HERMAN MYNTER then read a "Review of Surgical Cases treated in the Buffalo General Hospital from March to July, 1888." (See page 49.) The essayist also exhibited several patients on whom he had operated with very satisfactory results.

Discussion:

DR. PHELPS believed that the profession of Buffalo was indebted to Dr. Mynter for introducing several methods of operative surgery that were real advances. First, he would call attention to Thiersch's method of skin grafting. Dr. Phelps had used this procedure with marked and manifest benefit in a number of cases. Second, to Schede's aseptic blood-clot method; where, for example, in scraping away carious bone, the resulting cavity is allowed to fill with blood under antiseptic precautions. The clot afterwards organizes, and becomes ossified many times sooner than if the cavity were to fill up with granulations, etc.

Dr. Heath thought that the record presented was excellent. When operating for varicocele, he usually left one vein and extirpated the rest. He had never seen an attack of orchitis follow this method.

Dr. Hartwig spoke of Dr. Mynter's success at skin grafting, and related some experiments he had been making in transplanting animal skin to the surfaces of ulcers, etc. At present he was using rabbits, and would take pleasure later in reporting the completion and the ultimate results of his work.

Dr. W. W. Potter mentioned Hamilton's efforts at skin grafting. He believed that this distinguished surgeon was the first American to adapt the measure to the treatment of old ulcers. He viewed the essayist's results as very encouraging, especially those operations for the cure of cicatricial contractions, as these cases have long been a source of dismay to surgeons.

Dr. Mynter was surprised to hear Dr. Hartwig speak of his own work with rabbits in a manner intimating that it was novel and new. Nearly a year ago he (Dr. Mynter) published an article in *The Medical Press of Western New York*, describing a number of cases of transplantation of animal skin. In that article he reported successful results where the skin of both pigs and rabbits had been used. He did not find it necessary to kill the animal when removing the epidermis according to Thiersch's method, for he knew, from the personal experience of the hospital staff and several members of the medical class, that the removal of the strips of skin was painful only in a very slight degree.

Dr. F. S. Crego followed with a paper on "Neuralgia and its Treatment."

Discussion :

Dr. Stockton was always pleased to have a subject like neuralgia come up for discussion, for it baffles our efforts at treatment as often, perhaps, as any other common ailment. Neuralgia is frequently anomalous regarding its reaction towards drugs. Sometimes a single remedy is efficacious, in ordinary doses it may be, or we may have to saturate our patient. Again, we frequently have to cast about making combinations, etc., before we give relief. It always seemed strange to him that, in an otherwise apparently healthy body, we should have such severe nerve pain.

Dr. Mynter would not use antifebrin, as advised by Dr. Crego, but rather would substitute antipyrine. He also did not agree with Dr. Crego in the use of the Faradic current for the relief of neuralgia, in a case of *tic douloureux*, for instance. He believed that galvanism was more suitable, and was to be mildly applied.

Essayists for the September 4th meeting announced as Dr. W. D. Greene: subject, "Diphtheritic Croup;" and Dr. A. H. Briggs: subject, "The Influence of Location on Disease and the Death-Rate of our City."

Adjourned at 10.30 P. M.

